# The polycomb group protein BMI-1 inhibitor PTC-209 is a potent anti-myeloma agent alone or in combination with epigenetic inhibitors targeting EZH2 and the BET bromodomains

**DOI:** 10.18632/oncotarget.21909

**Published:** 2017-10-20

**Authors:** Mohammad Alzrigat, Alba Atienza Párraga, Muntasir Mamun Majumder, Anqi Ma, Jian Jin, Anders Österborg, Hareth Nahi, Kenneth Nilsson, Caroline A. Heckman, Fredrik Öberg, Antonia Kalushkova, Helena Jernberg-Wiklund

**Affiliations:** ^1^ Science for Life Laboratory, Department of Immunology, Genetics and Pathology, Rudbeck Laboratory, Uppsala University, Uppsala, Sweden; ^2^ Institute for Molecular Medicine Finland (FIMM), Helsinki Institute of Life Science, University of Helsinki, Helsinki, Finland; ^3^ Departments of Pharmacological Sciences and Oncological Sciences, Icahn School of Medicine at Mount Sinai, New York, NY, USA; ^4^ Department of Oncology-Pathology, Karolinska University Hospital, Solna, Stockholm, Sweden; ^5^ Department of Medicine, Unit of Hematology, Karolinska University Hospital, Huddinge, Stockholm, Sweden

**Keywords:** multiple myeloma, epigenetics, polycomb, BMI-1, PTC-209

## Abstract

Multiple myeloma (MM) is a tumor of plasmablasts/plasma cells (PCs) characterized by the expansion of malignant PCs with complex genetic aberrations in the bone marrow (BM). Recent reports, by us and others, have highlighted the polycomb group (PcG) proteins as potential targets for therapy in MM. The PcG protein BMI-1 of the polycomb repressive complex 1 (PRC1) has been reported to be overexpressed and to possess oncogenic functions in MM. Herein, we report on the anti-myeloma effects of the BMI-1 inhibitor PTC-209 and demonstrate that PTC-209 is a potent anti-myeloma agent *in vitro* using MM cell lines and primary MM cells. We show that PTC-209 reduces the viability of MM cells via induction of apoptosis and reveal that the anti-MM actions of PTC-209 are mediated by on-target effects i.e. downregulation of BMI-1 protein and the associated repressive histone mark H2AK119ub, leaving other PRC1 subunits such as CBX-7 and the catalytic subunit RING1B unaffected. Importantly, we demonstrate that PTC-209 exhibits synergistic and additive anti-myeloma activity when combined with other epigenetic inhibitors targeting EZH2 and BET bromodomains. Collectively, these data qualify BMI-1 as a candidate for targeted therapy in MM alone or in combinations with epigenetic inhibitors directed to PRC2/EZH2 or BET bromodomains.

## INTRODUCTION

Multiple myeloma (MM) is a genetically complex and heterogeneous disease characterized by abnormal proliferation of clonal plasmablasts/plasma cells (PCs) in the bone marrow (BM) [1–3]. Current treatment strategies in MM such as the use of proteasome inhibitors (e.g. bortezomib) and immunomodulatory drugs (e.g. thalidomide and lenalidomide) have improved the outcome of patients [4]. However, MM remains a therapeutically challenging malignancy due to the high rate of relapse and the development of drug resistance with a median survival time of less than 5 years [5]. The current view suggests that a combination of genetic and epigenetic aberrations accompanied by growth support from the tumor microenvironment in the bone marrow are important factors mediating drug resistance and relapse in MM [6, 7].

Epigenetic mechanisms play important roles in fundamental biological processes such as maintenance of pluripotency, cellular differentiation and cell reprogramming [8, 9], and aberrations in epigenetic modifiers and mediators have been implicated in tumor initiation and progression [10–12]. Supporting the importance of epigenetic mechanisms in MM pathogenesis, genetic alterations affecting components of epigenetic machineries i.e. epigenetic modifiers, mediators and substrates such as histone proteins, have been reported in MM [13–15]. In addition, deregulated expression and function of epigenetic modifiers such as the enhancer of zeste homolog 2 (EZH2) [16–18], the multiple myeloma SET domain containing-protein (MMSET) [19, 20] and members of the Jumonji C-domain-containing histone demethylases KDM6B [21] and KDM3A [22] are frequently observed in MM. Collectively, these data point towards a potential role of epigenetic reprogramming in MM, and highlight epigenetic modifiers as promising therapeutic targets in MM.

The polycomb group protein B Lymphoma Mo-MLV Insertion Region 1 (BMI-1) is a core subunit of the polycomb repressive complex 1 (PRC1), which plays important roles during normal development and tumorigenesis [23, 24]. PRC1 mediates gene repression via mono-ubiquitination of lysine 119 on histone H2A (H2AK119ub) and subsequent chromatin compaction [25, 26]. BMI-1 is involved in the regulation of important biological processes such as cell cycle, DNA damage response, stemness and senescence [27]. BMI-1 was initially discovered as a co-factor of c-MYC in lymphomagenesis [28, 29]. Later on, BMI-1 has been described as an oncogene in a wide range of solid and hematopoietic tumors [27, 30]. Notably, BMI-1 has been shown to mediate the growth and survival of cancer stem cells in solid and hematological malignancies [31–33]. Recently, the development of PTC-209, a small specific inhibitor of BMI-1, has opened new avenues to evaluate the therapeutic potential of BMI-1 in cancer including MM [31, 32, 34, 35]. BMI-1 was recently found to be overexpressed in MM compared to normal PCs [36–38] and to promote MM cells growth *in vitro* and *in vivo* [37]. Recent analyses of gene expression profiling in MM have revealed the overexpression of BMI-1 in all stages of MM progression as compared with normal bone marrow PCs [34]. In addition, high expression of BMI-1 was suggested as a predictor of poor survival in relapsed MM cases treated with bortezomib or dexamethasone [34]. Collectively, these data suggest an important role for BMI-1 in MM pathogenesis and response to treatment and highlight BMI-1 as a potential target for therapy.

In this study, we provide data further emphasizing BMI-1 as a potential therapeutic target in MM using PTC-209. We show that PTC-209 is indeed a potent anti-MM agent by reducing the viability of MM cell lines and primary MM cells from newly diagnosed or relapsed patients. We report that these effects are mediated by on-target effects by reducing the BMI-1 protein levels and the global level of the associated H2AK119ub. Importantly, PTC-209 did not affect other PRC1 subunits such as CBX-7 and, especially, the catalytic subunit RING1B. We also present PTC-209 as a promising combinatorial agent with specific epigenetic inhibitors targeting the polycomb group protein EZH2 and BET bromodomains.

## RESULTS

### The BMI-1 inhibitor PTC-209 is a potent anti-myeloma agent *in vitro*

**Figure 1:**
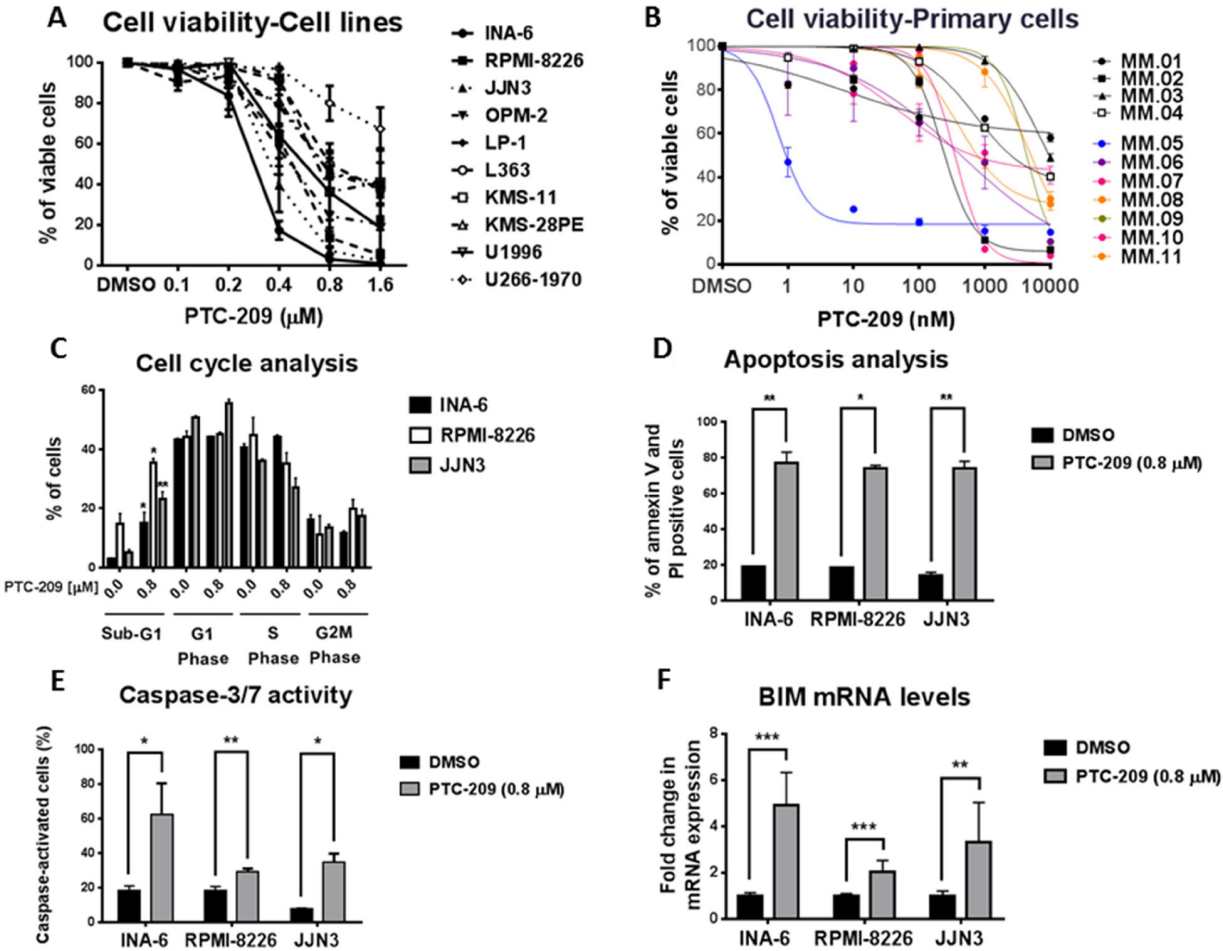
PTC-209 is a potent anti-myeloma agent that induces apoptosis (**A**) PTC-209 reduces the viability of MM cell lines. MM cells were treated with a range of PTC-209 concentrations for 48 hours. DMSO was used as control treatment. Cell viability was assessed using AlamarBlue assay. (**B**) PTC-209 reduces the viability of primary MM cells isolated from newly diagnosed or relapsed MM patients. Cell viability was represented as relative to DMSO-treated sample. Cell viability was measured by CellTiter-Glo assay 72 hours after treatment. (**C**–**F**) PTC-209 induces apoptosis in MM, which is evident by the accumulation of cells in sub-G1 phase (C) and an increase in the percentage of Annexin V and PI positive cells (D). PTC-209 mediated induction of apoptosis is further confirmed by the increase in caspase-3/7 activity (E) and induced expression of the pro-apoptotic gene BIM (F). Cell cycle was analyzed by measuring the DNA content using propidium iodide (PI) staining, while apoptosis was measured by assessing percentage of Annexin V and PI positive cells and caspase-3/7 activity. The impact of PTC-209 treatment on BIM expression was evaluated by RT-qPCR analysis of mRNA levels. Actin was used as the reference gene. Cells were treated for 48 hours with 0.8 µM PTC-209 or DMSO as control. Error bars represent standard deviation of three independent biological experiments from MM cell lines and three technical replicates for primary cells. *P*-values were calculated using student *t*-test in GraphPad Prism, *P*-value: * ≤ 0.05; ** ≤ 0.01; *** ≤ 0.001.

The reports on BMI-1 overexpression in MM and the development of PTC-209 as a specific inhibitor of BMI-1 prompted us to evaluate the therapeutic potential of targeting BMI-I by using PTC-209 in *in vitro* models of MM. To this end, we assessed the effects of PTC-209 treatment on the viability of MM cell lines and primary cells. PTC-209 exhibited a potent anti-myeloma activity, reducing the viability of MM cell lines at concentrations ranging up to 1.6 µM over 48 hours of treatment (Figure 1A). PTC-209-mediated reduction of cell viability was variable with INA-6 being the most responsive and U266-1970 being the least responsive cell line (Figure 1A). Notably, PTC-209 reduced the viability of MM cell lines tested in this study as early as 24 hours post-treatment (Supplementary Figure 1). To further investigate the anti-MM effects of PTC-209, we tested the effects on the viability of CD138^+^ malignant PCs purified from newly diagnosed (patients 1–4) or relapsed patients (patients 5–11) in response to 72 hours treatment with a range of PTC-209 concentrations (Figure 1B). We found that a high concentration (10 µM) of PTC-209 reduced the viability of all primary MM cells (Figure 1B). Interestingly, the response of malignant CD138^+^ PCs to 1 µM of PTC-209 identified 3 groups of patients: non-responsive, moderate responders and high responders (Figure 1B). The effect of PTC-209 in reducing the viability of primary MM cells in this study was independent of the disease diagnosis i.e. newly diagnosed or refractory as well as the cytogenetic karyotype of the patients (Supplementary Table 1).

### PTC-209 induces apoptosis in MM cells

To further decipher the anti-MM potential of PTC-209, we evaluated the effects of PTC-209 treatment (0.8 µM) for 48 hours on cell cycle and apoptosis induction by flow cytometry in three of the MM cell lines: INA-6, RPMI-8226 and JJN3. Cell cycle analysis revealed that PTC-209 treatment did not affect the distribution of the cells in different phases of the cell cycle, but induced a significant increase in the percentage of apoptotic cells in the sub-G1 phase (Figure 1C and Supplementary Figure 2). The increase in the percentage of sub-G1 apoptotic cells upon 0.8 µM treatment with PTC-209 varied between cell lines i.e. 5-fold in INA-6 (3 to 15%), 2.5-fold in RPMI-8226 (14 to 35%) and 4-fold in JJN3 (5–23%). To specifically study markers of apoptosis, we evaluated by flow cytometry Annexin V and PI positive cells in control DMSO or PTC-209 (0.8 µM) treated MM cell lines. We found that PTC-209 significantly increased the percentage of Annexin V and PI positive MM cells as compared to DMSO control treatment (Figure 1D and Supplementary Figure 3). The increase in Annexin V/PI-positive cells due to treatment with PTC-209 varied between cell lines i.e. 4-fold in INA-6 (19.2 to 77.7%), 4-fold in RPMI-8226 (18.3 to 73.8%) and 5-fold in JJN3 (14.2 to 74.1%). We further confirmed the apoptotic effects of PTC-209 in MM cell lines by showing increased caspase-3/7 activity (Figure 1E) and induced expression of the pro-apoptotic gene BIM at the mRNA level (Figure 1F).

### PTC-209 demonstrates on-target effects in MM

**Figure 2:**
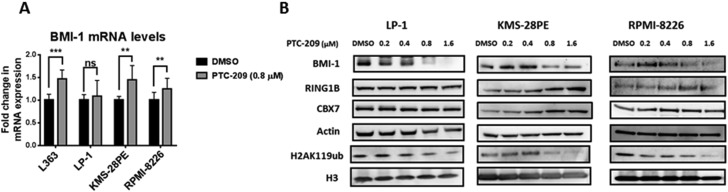
PTC-209 demonstrates on-target effects in MM via downregulation of BMI-1 protein and H2AK119ub levels (**A**) PTC-209 treatment does not reduce the BMI-1 transcript levels. Analysis of BMI-1 mRNA levels by RT-qPCR in MM cell lines. Cells were treated with DMSO or 0.8 µM of PTC-209 for 48 hours. Changes in mRNA expression were represented relative to DMSO-treated cells. Actin was used as reference gene. Error bars represent standard deviation of three independent biological experiments. *P*-values were calculated using student *t*-test in GraphPad Prism, *P*-value: * ≤ 0.05; ** ≤ 0.01; *** ≤ 0.001. (**B**) PTC-209 treatment reduces BMI-1 protein and H2AK119ub global levels in MM cell lines. Analysis of PTC-209 impact on protein levels of PRC1 subunits: BMI-1, CBX-7, RING1B and the associated repressive histone mark H2AK119ub in MM cell lines. Cells were treated with a range of PTC-209 concentrations for 48 hours. DMSO was used as control treatment. Actin and total histone H3 were used as loading controls. Western blots are representative of three independent biological experiments.

PTC-209 was initially selected for its capacity to reduce BMI-1 protein expression and to subsequently inactivate PRC1 activity as represented by a decrease in the global levels of H2AK119ub without affecting other subunits of the PRC1 complex [31]. To investigate the on-target effects of PTC-209 in MM, we evaluated the impact of PTC-209 treatment on BMI-1 expression and the global levels of H2AK119ub in several MM cell lines. By reverse transcription quantitative real-time PCR (RT-qPCR) we found that PTC-209 treatment did not reduce the expression of BMI-1 at the mRNA levels, but rather led to an increase in BMI-1 transcript levels in most of the MM cell lines tested in this study (Figure 2A). Importantly, western blot analysis of BMI-1 protein revealed that PTC-209 treatment downregulated BMI-1 protein levels (Figure 2B). The downregulation of BMI-1 protein as a consequence of PTC-209 treatment was accompanied by a reduction in the global levels of the H2AK119ub histone mark mediated by the PRC1 complex (Figure 2B). We further documented the on-target effects of PTC-209 in MM by showing that other PRC1 subunits i.e. CBX-7 and, most importantly, the E3 ubiquitin ligase RING1B were not affected by the PTC-209 treatment (Figure 2B).

### PTC-209 demonstrates synergistic and additive activity with UNC1999 and JQ1 in MM cell lines

**Figure 3:**
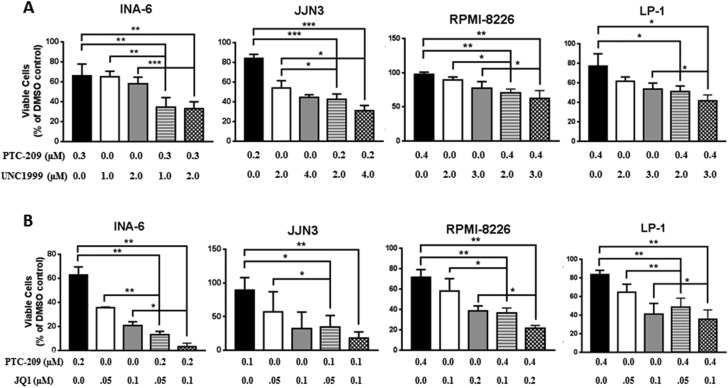
PTC-209 shows synergistic and additive effects with UNC1999 and JQ1 in MM cell lines Synergistic and/or additive activity of drug combinations was confirmed by co-treatment of MM cell lines with PTC-209 and either UNC1999 (**A**) or JQ1 (**B**) at varying concentrations. Combination index (CI) values were determined using CompuSyn software. CI values < 0.8; 0.8–1.2 or > 1.2 indicated synergistic, additive, or antagonistic drug activities, respectively. DMSO was used as control treatment. Combination treatments were performed for 72 hours. Error bars represent standard deviation of three independent biological experiments. *P*-values were calculated using student *t*-test in GraphPad Prism, *P*-value: * ≤ 0.05; ** ≤ 0.01; *** ≤ 0.001.

Having shown potent anti-myeloma activity for PTC-209, we sought to investigate whether PTC-209 represents a potent anti-MM agent in combination with other epigenetic inhibitors. The possibility of inhibiting both polycomb repressive complexes i.e. PRC1 and PRC2 is highly attractive and supported by the growing evidences showing possible collaboration between the complexes in gene silencing [39–41]. In addition, we and others have recently demonstrated that targeted inhibition of the PRC2 enzymatic subunit EZH2 by highly selective inhibitors had anti-myeloma effects [42–46]. To this end, we combined PTC-209 with the EZH2 inhibitor UNC1999 and assessed the effects of single or combinatorial treatments on the viability of the MM cell lines INA-6, JJN3, RPMI-8226 and LP-1. We found that combinations of PTC-209 and UNC1999 induced a significant reduction in cell viability when compared to single agent treatment in all cell lines tested in this study as shown in Figure 3A for some selected combinations investigated in this study. Using CompuSyn software, we found that combination treatments of PTC-209 and UNC1999 for 72 hours had synergistic effects (combination index (CI) < 0.8) in the MM INA-6 cell line at all concentrations tested (Table 1). In the MM cell line JJN3, we found that combinations of PTC-209 and UNC1999 demonstrated both synergistic (CI < 0.8) and additive (CI between 0.8–1.2) effects (Table 1). PTC-209 and UNC1999 combinations had mainly additive effects (CI between 0.8–1.2) in the RPMI-8226 and LP-1 cell lines, with some combinations showing slight antagonistic effects (Table 1). The recent finding by Bolomsky et al. [34] showing that PTC-209 reduced the expression of MM survival genes such as c-MYC urged us to evaluate the efficacy of PTC-209 as anti-MM agent also in combination with the BET bromodomains inhibitor JQ1. We found that the combined treatment of PTC-209 and JQ1 induced a significant reduction in cell viability over single agent treatment in INA-6, JJN3, RPMI-8226 and LP-1 MM cell lines (Figure 3B). Combinations of PTC-209 and JQ1 demonstrated synergistic effects (CI < 0.8) in the INA-6 cell line (Table 2). In JJN-3 and RPMI-8226 PTC-209 and JQ1 combinations showed synergistic (CI < 0.8) and additive (CI between 0.8–1.2) anti-MM effects, while mainly additive effects (CI between 0.8–1.2) were observed in the MM cell line LP-1 (Table 2).

**Table 1:** PTC-209 and UNC1999 combination treatments, inhibition rate and combination index (CI)

Cell Line	PTC-209 (µM)	UNC1999 (µM)	Inhibition Rate	Combination Index (CI)
INA-6	0.1	1	0.44415	0.57996
	0.1	2	0.49388	0.64551
	0.2	1	0.46436	0.68419
	0.2	2	0.50582	0.75362
	0.3	1	0.65551	0.38245
	0.3	2	0.66991	0.40216
JJN3	0.1	2	0.47870	1.22874
	0.1	4	0.78551	0.39410
	0.2	2	0.57284	1.09227
	0.2	4	0.68961	0.92479
	0.4	2	0.76196	1.14930
	0.4	4	0.94817	0.69431
RPMI-8226	0.2	2	0.19562	1.11126
	0.2	3	0.33201	1.13197
	0.4	2	0.29207	1.26375
	0.4	3	0.37471	1.37317
	0.6	2	0.79527	0.86478
	0.6	3	0.81141	0.94670
LP-1	0.2	2	0.50284	0.94805
	0.2	3	0.50177	1.23164
	0.4	2	0.48820	1.39678
	0.4	3	0.58624	1.24663
	0.6	2	0.75672	0.93147
	0.6	3	0.77391	0.95192

Combination index (CI) values were determined using CompuSyn software (ComboSyn, Inc., Paramus, NJ). CI values < 0.8; 0.8–1.2 or > 1.2 indicated synergistic, additive, or antagonistic drug activities, respectively.

**Table 2:** PTC-209 and JQ1 combination treatments, inhibition rate and combination index (CI)

Cell Line	PTC-209 (µM)	JQ1 (µM)	Inhibition Rate	Combination Index (CI)
INA-6	0.1	0.05	0.91980	0.28170
	0.1	0.1	0.92099	0.40982
	0.2	0.05	0.86716	0.63779
	0.2	0.1	0.96916	0.27303
	0.3	0.05	0.95350	0.41319
	0.3	0.1	0.93535	0.60423
JJN3	0.1	0.05	0.65742	0.69484
	0.1	0.1	0.82372	0.67699
	0.2	0.05	0.56127	1.11905
	0.2	0.1	0.79496	0.86991
	0.4	0.05	0.73771	0.91708
	0.4	0.1	0.81171	1.00260
RPMI-8226	0.2	0.1	0.52490	1.13203
	0.2	0.2	0.73503	0.92359
	0.4	0.1	0.63731	1.20221
	0.4	0.2	0.78667	1.03513
	0.6	0.1	0.94489	0.52199
	0.6	0.2	0.96883	0.43832
LP-1	0.2	0.05	0.45307	1.10495
	0.2	0.1	0.62035	1.17057
	0.4	0.05	0.51424	1.25665
	0.4	0.1	0.64495	1.32982
	0.6	0.05	0.80423	0.71373
	0.6	0.1	0.84470	0.80021

Combination index (CI) values were determined using CompuSyn software (ComboSyn, Inc., Paramus, NJ). CI values < 0.8; 0.8–1.2 or > 1.2 indicated synergistic, additive, or antagonistic drug activities, respectively.

### PTC-209 has synergistic and additive activity with UNC1999 and JQ1 in primary MM cells

**Figure 4:**
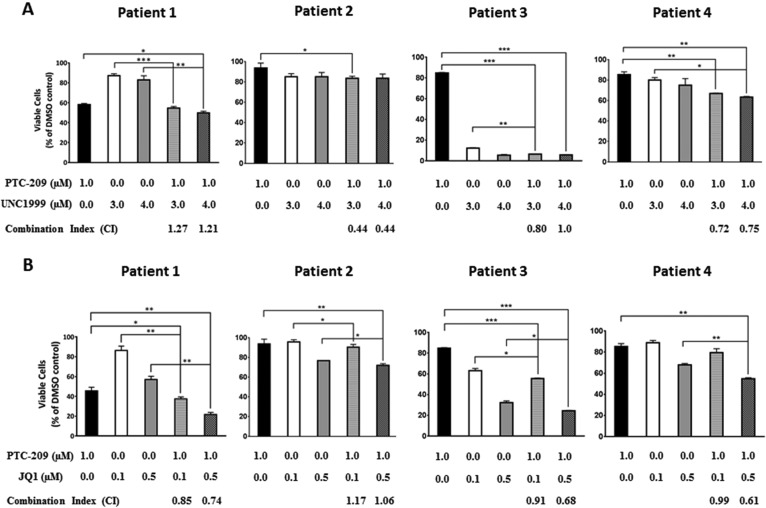
PTC-209 shows synergistic and additive effects with UNC1999 and JQ1 in patient derived-MM cells Synergistic and/or additive activity of drug combinations was confirmed by co-treatment of CD138^+^ cells purified from newly diagnosed MM patients with PTC-209 and either UNC1999 (**A**) or JQ1 (**B**). Combination index (CI) values were determined using CompuSyn software. CI values < 0.8; 0.8–1.2 or > 1.2 indicated synergistic, additive, or antagonistic drug activities, respectively. DMSO was used as control treatment. Combinatorial treatments were performed for 72 hours. Error bars represent standard deviation of three technical replicates. *P*-values were calculated using student *t*-test in GraphPad Prism, *P*-value: * ≤ 0.05; ** ≤ 0.01; *** ≤ 0.001.

Having shown that PTC-209 possesses synergistic and additive effects when combined with UNC1999 or JQ1 in MM cell lines, we sought to investigate whether PTC-209 combinatorial effects with UNC1999 or JQ1 could be recapitulated on CD138^+^ primary cells purified from MM patients. Indeed we found that combinations of PTC-209 with either UNC1999 (Figure 4A) or JQ1 (Figure 4B) reduced the viability of primary MM cells compared with single agent treatment. Similar to combinations in MM cell lines, we observed that combinations of PTC-209 with UNC1999 or JQ1 had synergistic (CI < 0.8) and additive (CI between 0.8–1.2) effects when combined on primary MM cells.

## DISCUSSION

Despite encouraging reports that current treatment strategies can improve multiple myeloma (MM) management, the disease remains fatal [1–4]. Therefore, identification of new therapeutic strategies is imperative for improved MM treatment. Recently, we and others have revealed the importance of epigenetic mechanisms as contributing factors in the development of MM [18, 37, 42, 47–53]. The recent success in the development of selective small-molecule inhibitors targeting epigenetic modifiers may now have the potential to increase our understanding of aberrant epigenetic gene regulation in cancer and how inhibiting these components could be utilized for future treatment of patients including MM [54, 55]. Among epigenetic modifiers, the BMI-1 protein of the PRC 1 complex now represents a promising target for therapy in MM. This notion is partly based on the fact that BMI-1 is overexpressed in MM during disease progression and relapse [34, 36–38]. Underlining the clinical relevance, BMI-1 overexpression was also found to correlate with poor outcome in refractory MM patients treated with bortezomib or dexamethasone [34]. In 2010, Jagani et al. reported on the importance of BMI-1 for MM growth *in vitro* and *in vivo* by shRNA-mediated knockdown of BMI-1 [37]. The recent development of PTC-209, a selective small-molecule inhibitor of BMI-1, has initiated the possibility to evaluate the therapeutic potential of BMI-1 in tumors including MM [31].

In this study we evaluated the anti-myeloma activity of PTC-209 on a panel of authenticated MM cell lines. We found that PTC-209 demonstrates potent anti-myeloma effects in all MM cell lines used in this study. We showed that PTC-209 induced apoptosis in MM cell lines, as determined by an increase in the percentage of Annexin V/PI positive cells and caspase-3/7 activity. This is in agreement with the findings of Bolomsky et al. [34]. Previously, BMI-1 has been suggested to support MM cell growth via direct repression of the pro-apoptotic gene BIM and BMI-1 knockdown reactivated the expression of BIM resulting in MM cell death [37]. In the present study, we evaluated the impact of PTC-209 on the expression of BIM and found that PTC-209 indeed increased the levels of BIM transcripts 48 hours post-treatment in MM cell lines. BIM upregulation was detected as early as 12 hours post-treatment with PTC-209, which coincided with BMI-1 protein downregulation, in the RPMI-8226 cell line (data not shown). This is in contrast to Bolomsky et al. who have reported that PTC-209 had no impact on BIM expression and suggested that PTC-209-mediated apoptosis in MM was likely a consequence of the upregulation of NOXA and downregulation of the anti-apoptotic MCL-1 [34]. We speculate that the discrepancy in the results concerning the impact of PTC-209 treatment on BIM expression between our study and Bolomsky et al. [34] might be due to the differences in the choice of time points at which BIM expression was evaluated. The data presented in this study demonstrates that PTC-209-induced apoptosis in MM may partly be mediated by direct upregulation of the pro-apoptotic gene BIM. This is in accordance with Jagani et al. showing that BIM knockdown may protect MM cells from the anti-MM phenotype mediated by BMI-1 knockdown [37]. In addition, our current study also explores the anti-myeloma activity of PTC-209 on primary MM cells isolated from both newly diagnosed and relapsed patients. Notably, we found that PTC-209 treatment reduced the viability of the majority of MM primary cells derived from both newly diagnosed and relapsed patients. Resistance of cancer cells to treatment and subsequent disease relapse have in some tumors been attributed to a small population of cells with stemness properties [56–58]. Interestingly, BMI-1 expression was found to be upregulated in relapsed MM [34]. As BMI-1 has been assigned an important role in conferring stemness to tumor cells in various types of cancer [31–33], we suggest that targeting BMI-1 in relapsed cancer patients including MM could represent a potential therapeutic strategy. The possibility that BMI-1 would confer stemness characteristics to MM cells is an interesting concept that warrants further investigation.

PTC-209 was initially found to reduce the BMI-1 expression in the human colorectal HCT116 cell line [31]. In this study, we showed that PTC-209 treatment in MM resulted in the downregulation of BMI-1 protein, but not downregulation of transcript levels. This is in agreement with the recent finding showing that PTC-209 did not affect BMI-1 transcript levels but downregulated BMI-1 protein levels in biliary tract cancer cells [32]. Our data thus suggest that PTC-209 regulates BMI-1 post-transcriptionally. Recent findings using PTC596 to inhibit BMI-1 have suggested protein degradation pathways as underlying mechanisms of drug action [59]. In this study, we found that proteasome inhibition using MG132 in RPMI-8226 and LP-1 cell lines did not counteract PTC-209 phenotype i.e. rescuing the BMI-1 protein levels and cell viability (data not shown), which may indicate that PTC-209 is an inhibitor of BMI-1 translation. We further investigated the on-target effects of PTC-209 in MM and evaluated the impact of PTC-209 on other subunits of the PRC1 complex. We could show that PTC-209 neither affected the enzymatic E3 ubiquitin ligase RING1 subunit nor other core subunits such as CBX-7. PTC-209-mediated downregulation of BMI-1 led to global reduction in the H2AK119ub histone mark, consistent with reduced ubiquitin ligase activity of PRC1. These data are in agreement with the initial finding reporting BMI-1 to be a target of PTC-209 [31]. The demonstration of on-target effects of PTC-209 in MM thus suggests chromatin-mediated mechanisms as important mediators underpinning its anti-MM activity.

Recently, PTC-209 was shown to have synergistic and/or additive effects when combined with dexamethasone, pomalidomide and carfilzomib in MM [34]. Supporting the use of pharmacological inhibition of BMI-1 in combinatorial regimens, shRNA-mediated knockdown of BMI-1 was found to sensitize MM cells to bortezomib treatment [60]. To further study the potential use of PTC-209 as an anti-MM agent, we investigated the anti-MM effects of PTC-209 in combinatorial epigenetic regimens in MM cell lines and malignant CD138^+^ primary cells. PRC1 and PRC2 co-operation in gene silencing has been suggested during normal development and tumorigenesis [39–41]. We and others have recently shown that EZH2 inhibition demonstrates anti-MM effects using *in vitro* and *in vivo* models [42–46]. Therefore, combinatorial inhibition of PRC1 and PRC2 is indeed of interest in MM. We used PTC-209 and the EZH2 inhibitor UNC1999 to assess the effects of single and combinatorial treatments on the viability of MM cell lines and primary cells *in vitro*. We found that combinations of PTC-209 and UNC1999 have synergistic and/or additive effects in reducing the viability of MM cells. Recently, PTC-209 was shown to downregulate the expression of MM survival genes such as c-MYC, MCL-1 and CCND1 [34]. Deregulation of c-MYC has been suggested to be an important step in disease progression from the asymptomatic monoclonal gammopathy of undetermined significance (MGUS) stage to the symptomatic MM in human [61] and in murine models [62]. Furthermore, targeted inhibition of c-MYC and its expression signature by BET bromodomains inhibitors has been proven to be a potential therapeutic strategy in MM [63–65]. Taken together, these findings prompted us to evaluate the combination of PTC-209 and JQ1 on the viability of MM cell lines. Indeed, we found that PTC-209 and JQ1 demonstrated synergistic and/or additive effects when combined in MM cell lines and primary cells. Collectively, the data presented in this study further support BMI-1 as a candidate for targeted therapy in MM, but also propose combinatorial epigenetic therapy as a novel treatment in MM.

In conclusion, the present study further emphasizes that the BMI-1 inhibitor PTC-209 has potent anti-MM activity and supports the notion that BMI-1 may be a potential therapeutic target in novel MM treatment strategies. We present epigenetic inhibitors as a novel therapeutic intervention for MM by further highlighting the potential use of BMI-1 inhibitors alone or in combination with other epigenetic inhibitors directed to PRC2/EZH2 or BET bromodomains in targeting the malignant MM cell.

## MATERIALS AND METHODS

### Cell culture and treatment

All MM authenticated cell lines [66] used in this study were maintained in RPMI-1640 AQmediaTM (Sigma) supplemented with 10% FBS (Sigma), 1% GlutaMax™ (Gibco) and antibiotics (penicillin 100 U/mL and streptomycin 50 mg/mL; Sigma) at 37°C in a humidified 5% CO_2_ in-air atmosphere. The INA-6 and U1996 cell lines were cultured in the presence of Interleukin-6 (IL-6). Exponentially growing cells were seeded at 200 000 cells/mL overnight before addition of reagents.

### Reagents

The BMI-1 inhibitor PTC-209 was purchased from (Sigma Aldrich, CAS #: 315704-66-6). The BET bromodomain inhibitor JQ1 was purchased from (BPS Bioscience, Catalog #: 27400). UNC1999 was synthesized according to previously published procedures [67].

### Cell viability assay for MM cell lines

MM cell lines were seeded in 12-well plate at 200 000 cells/ml overnight then treated with a range of PTC-209 concentrations for 48 hours with DMSO used as control treatment. On the day of analysis, cells were seeded in triplicate wells in 96-well flat-bottom plates. Cell viability was assessed using Resazurin assay reduction method using AlamarBlue (Sigma-Aldrich) as previously described [18]. Fluorescence level of at least 5 times of the blank was set as threshold for DMSO control treated sample to be included in the analysis.

### Patients and patient material

The ethics committees of the participating hospitals approved the study and patient samples obtained following informed consent in compliance with the Declaration of Helsinki. A total of 11 bone marrow (BM) aspirates were collected from 4 diagnostic and 7 relapsed myeloma patients. Patient characteristics are detailed in Supplementary Table 1. For drug combination experiments, heparinized bone marrow samples were obtained from newly diagnosed MM patients in accordance with the Declaration of Helsinki and approved by the local ethics committees of Uppsala and Stockholm (Dnr 2004:M-332 and 2010/1478-32).

### Cytogenetics

Plasma cells were selected by immunomagnetic enrichment for CD138^+^ cells (Human Whole Blood CD138 Microbeads Column Kit, Miltenyi Biotec, Bergisch Gladbach, Germany). Selected cells (*n* ≥ 100) were used for interphase fluorescence *in situ* hybridization (FISH) following the guidelines of the European Myeloma Network 2012 [68]. The probes used for FISH were described previously [69].

### Drug sensitivity and resistance testing

CD138^+^ cells were enriched with the EasySep™ Human CD138 Positive Selection Kit (StemCell Technologies, Grenoble, France) using the mononuclear cell fraction of BM aspirates following gradient separation (Ficoll-Paque PREMIUM; GE Healthcare, Little Chalfont, Buckinghamshire, UK). CD138^+^ cells derived from myeloma patients with 90% purity were tested against PTC-209 in 5 concentrations in 10-fold dilutions covering a 10,000-fold concentration range (1–10,000 nM). In brief, 5 µL of a 5× PTC-209 solution was diluted in cell culture medium comprised of RPMI-1640 medium supplemented with 10% fetal bovine serum, 2 mM L-glutamine, penicillin (100 U/mL), streptomycin (100 µg/mL) and 25% conditioned medium from the HS-5 human bone marrow stromal cell line [70] and added to 384-well drug plates. CD138^+^ cells were diluted in the cell culture medium and 20 µL of the cell suspension containing 5000 cells was transferred to each well using a MultiDrop Combi peristaltic dispenser (Thermo Scientific, Waltham, MA, USA). The plates were incubated in a humidified environment at 37°C and 5% CO_2_. Cell viability was measured after 72 h using the CellTiter-Glo assay (Promega, Madison, WI, USA) with a PHERAstar^®^ microplate reader to measure luminescence (BMG-Labtech, Offenburg, Germany). The data was normalized to untreated cells (DMSO only). Results from triplicate screens were used to generate 5-point dose-response curves in Graph Pad Prism version 7.00 for MAC OS, GraphPad Software, La Jolla California USA.

### Flow cytometric analysis

Cells were seeded at 200 000 cells/mL overnight before addition of reagents then were cultured for 48 hours in the presence of DMSO control or 0.8 µM of PTC-209. Apoptosis was quantified by Annexin V (AV)-fluorescein isothocyanate (FITC) using TACS Annexin V-FITC Apoptosis Kit (R&D Systems, Gaithersburg, MD, USA). Samples were treated according to manufacturer’s recommendations and analyzed by flow cytometry (FACScan), presenting apoptotic cells as Annexin V-positive/PI-negative cells and necrotic cells as Annexin V-positive/PI-positive cells. Cell cycle analysis was performed by quantitation of DNA content using propidium iodide (PI) DNA staining. The distribution of cells in the different cycle phases was performed following the Vindelov method. In short, cells were washed with ice-cold PBS, lysed using NP-40 buffer and trypsin (0.03 mg/mL) for 10 minutes at room temperature. Next, samples were incubated in trypsin inhibitor (0.5 mg/mL) and ribonuclease A (0.1 mg/mL) for 10 minutes at room temperature. DNA staining was performed by addition of PI (0.42 mg/mL) and incubation for 15 minutes at 4°C. The modfit LT 3.1 Analysis Software (Verity Software House) was used to calculate the proportion of cycling cells in each of the cell cycle phases.

### Protein extraction and western blot

Following treatment with DMSO or PTC-209 for 48 hours, MM cell lines were harvested, washed with ice-cold PBS and collected at 1500 *rpm* for 5 minutes. Total cellular protein was extracted using RIPA extraction buffer with freshly added protease inhibitor cocktail. Western blotting was performed as previously described [42]. Histone proteins were extracted using the Episeeker histone extraction kit (abcam, Ab113476) following the manufacturer’s procedure. Antibodies against BMI-1 (Santa Cruz, sc-390443), RING1B (ab3832), CBX-7 (abcam, ab21873), Actin (Santa Cruz, sc-1616), H2AK119ub (Millipore) and Histone H3 (abcam, Ab1791) were used in this study.

### mRNA expression analysis

Total RNA was extracted using TRIzol^®^ reagent (Invitrogen) and reverse transcription was performed using random primers (Invitrogen) was performed on 1 µg of total RNA using SuperScriptTM III Reverse Transcriptase (Invitrogen) according to the manufacturer’s protocol. RT-qPCR analysis of mRNA was performed using TaqMan^®^ gene expression assays (Applied Biosystems) for BMI-1 (Assay ID: Hs00995536_m1, Catalog #: 4448892), BIM (Assay ID: Hs00708019_s1, Catalog #: 4453320) and Actin (Assay ID: Hs01060665_g1, Catalog #: 4448892)as housekeeping mRNA.

### Combination treatment and calculation of combination indexes

The MM cell lines were seeded at 200 000 cells/ml in 12 well plates overnight before treatment. The INA-6 cell line was cultured in the presence of IL-6. Cells were treated with single drugs or combination of drugs for 72 hours. For primary cells, CD138^+^ MM cells were purified from 4 newly diagnosed MM patients using immunomagnetic purification by Whole Blood Column Kit (MACS, Miltenyi Biotec, Paris, France) according to the manufacturer’s protocols. Subsequently, the purity of the CD138-enriched fraction was evaluated by May-Grünwald-Giemsa staining (Supplementary Table 2). Cell viability was assessed using AlamarBlue assay (Sigma-Aldrich) as previously described [18] and as mentioned above. Combination index (CI) was calculated using CompuSyn software (ComboSyn, Inc., Paramus, NJ). CI values < 0.8; 0.8–1.2 or >1.2 indicated synergistic, additive, or antagonistic drug activities, respectively.

### Statistical analysis

Paired, two-tailed Student *t*-test was calculated using GraphPad Prism, *: *P*-value ≤ 0.05, **: *P*-value ≤ 0.01, ***: *P*-value ≤ 0.001.

## SUPPLEMENTARY MATERIALS FIGURES AND TABLES


