# Supplementary material for: The polycomb group protein BMI-1 inhibitor PTC-209 is a potent anti-myeloma agent alone or in combination with epigenetic inhibitors targeting EZH2 and the BET bromodomains
Source: Oncotarget. 2017 Oct 20;8(61):103731–43. doi: 10.18632/oncotarget.21909 (PMC5732762; doi:10.18632/oncotarget.21909)
Supplement: Supplementary file 1 [file oncotarget-08-103731-s001.pdf]

The polycomb group protein BMI-1 inhibitor PTC-209 is a potent anti-myeloma agent alone or in combination with epigenetic inhibitors targeting EZH2 and the BET bromodomains

SUPPLEMENTARY MATERIALS

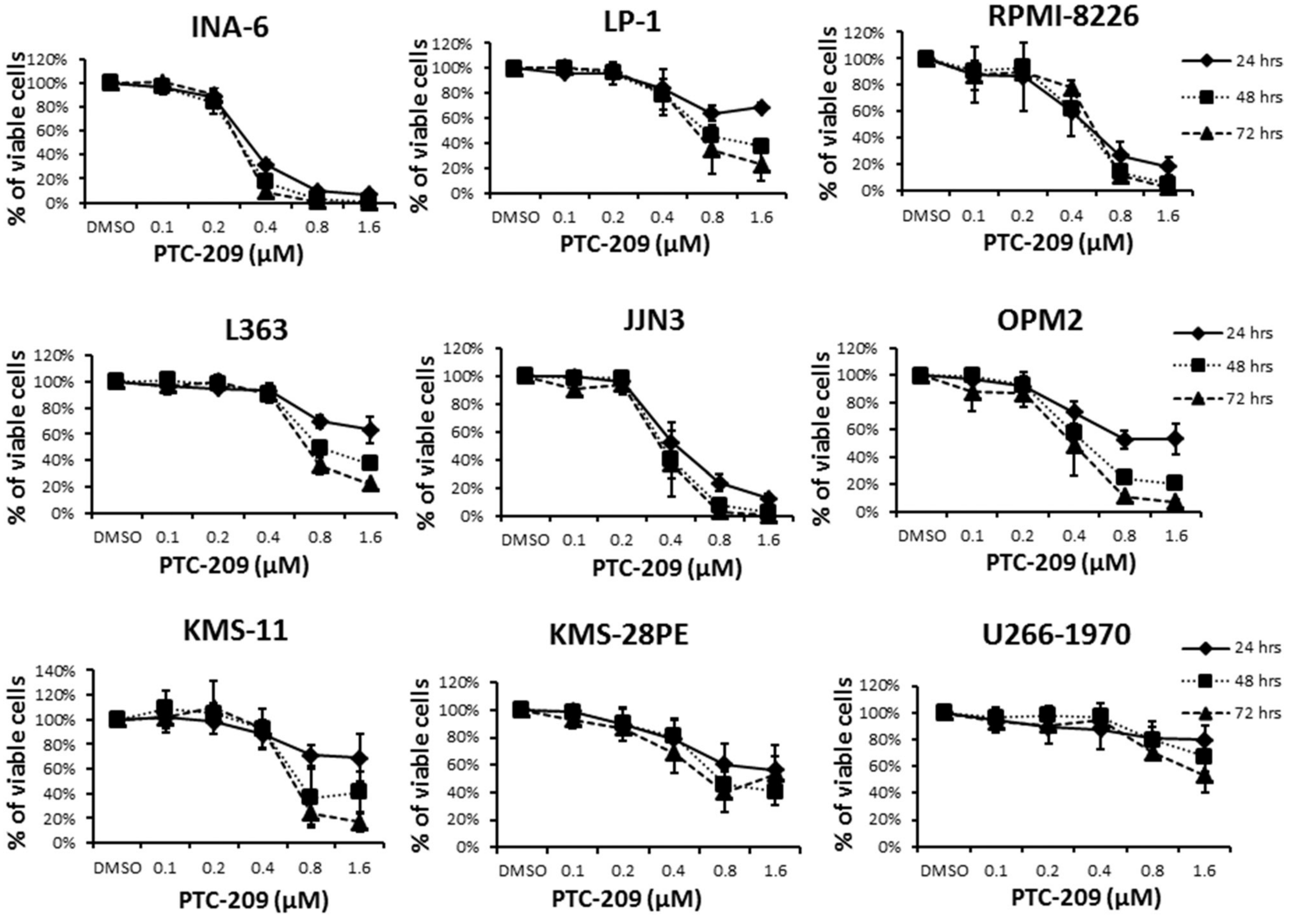

Supplementary Figure 1: PTC-209 reduces the viability of MM cell lines. MM cells were treated with a range of PTC-209 concentrations and cell viability was assessed 24, 48 and 72 hours post-treatment. DMSO was used as control treatment. Cell viability was assessed using AlamarBlue assay. Error bars represent standard deviation of three independent biological experiments.

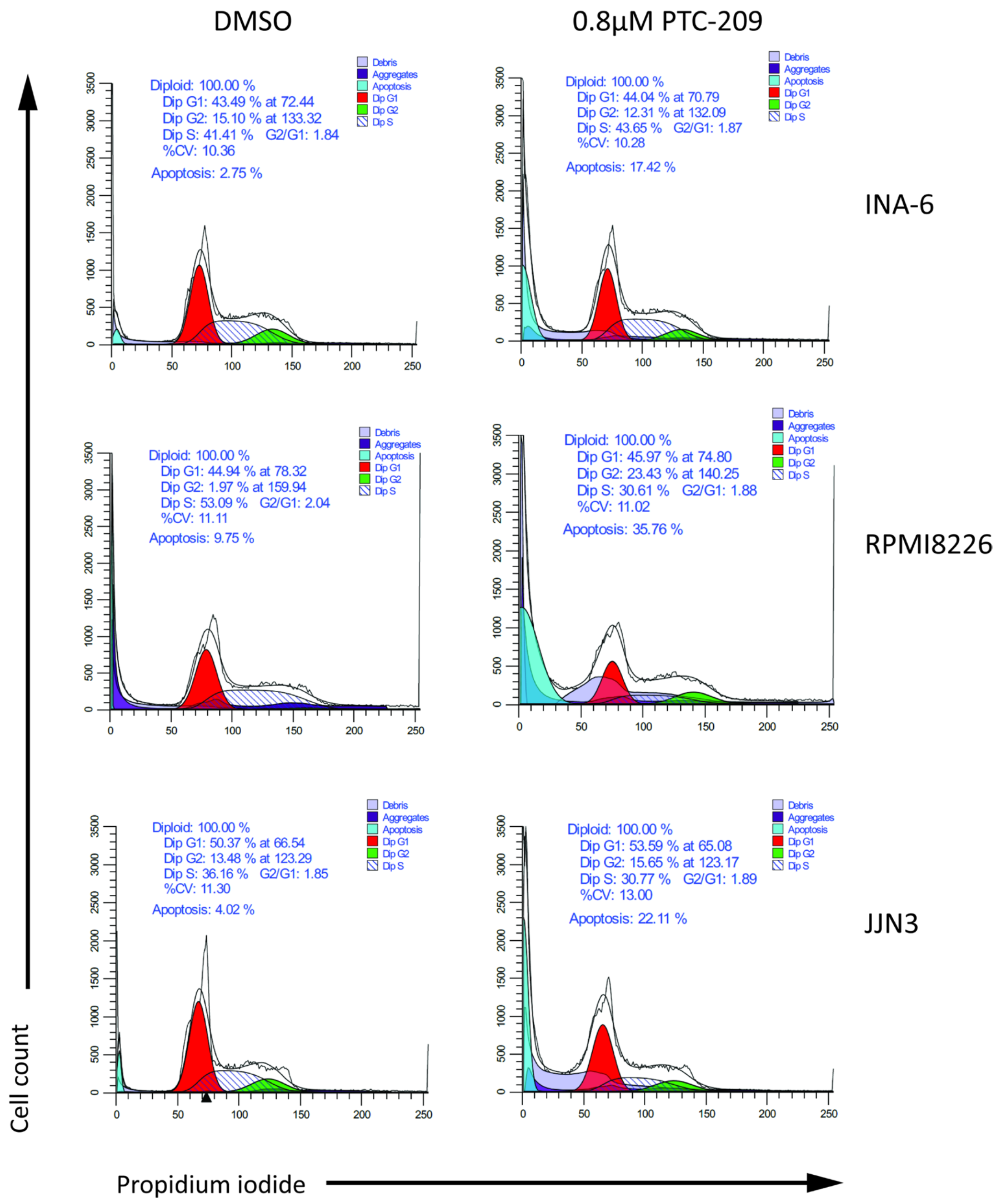

Supplementary Figure 2: PTC-209 treatment induces the accumulation of cells in the sub-G1 phase. The MM cell lines INA-6, RPMI-8226 and JJN3 were treated with 0.8 µM of PTC-209 for 48 hours and cell cycle was analyzed by FACS analysis by measuring the DNA content using propidium iodide (PI) staining. DMSO was used as control treatment. The modfit LT 3.1 Analysis Software (Verity Software House) was used to calculate the proportion of cycling cells in each of the cell cycle phases. The histograms are representative of three independent biological experiments.

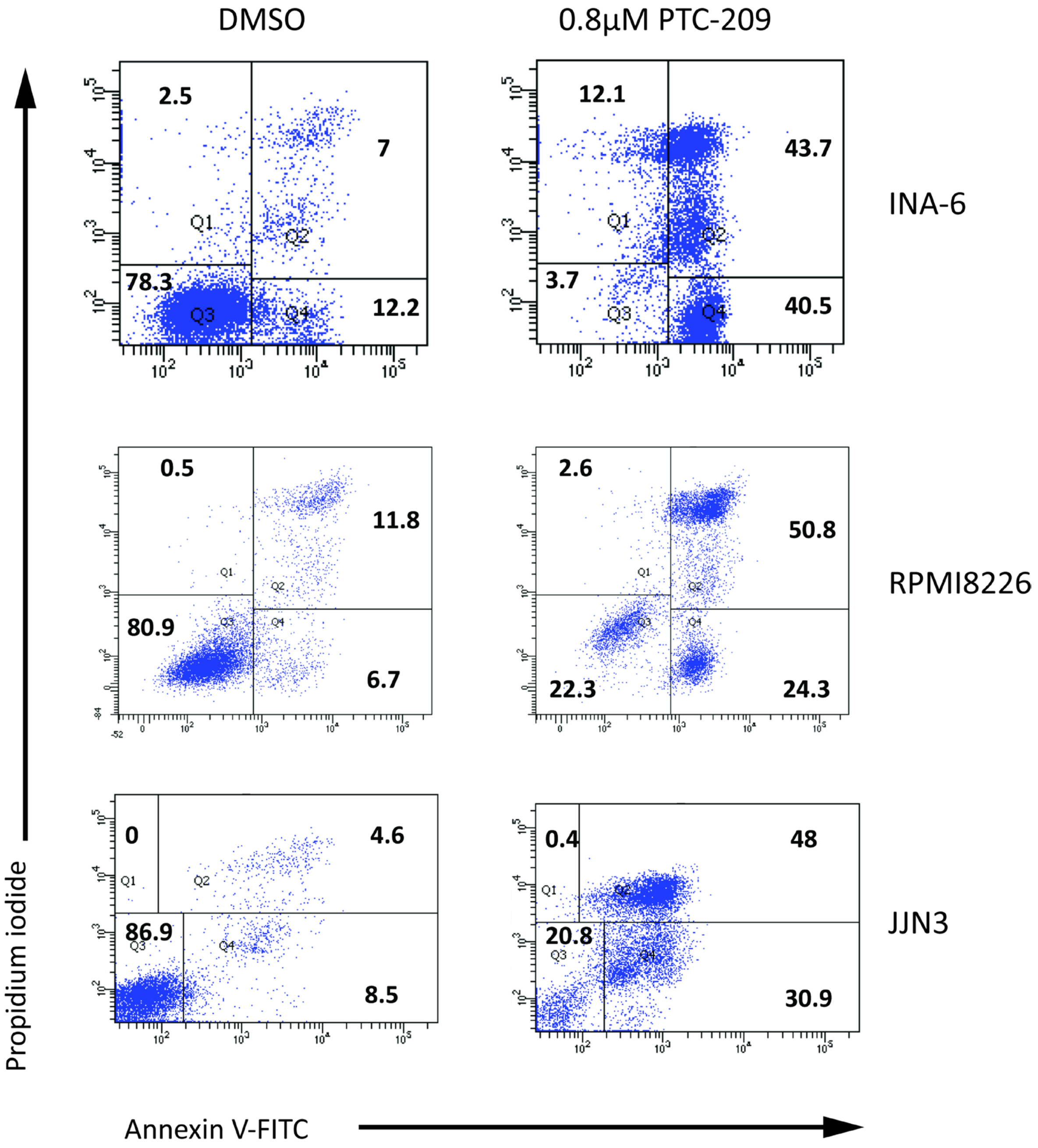

Supplementary Figure 3: PTC-209 induces apoptosis in MM cell lines. The MM cell lines INA-6, RPMI-8226 and JJN3 were treated with 0.8 µM of PTC-209 for 48 hours and apoptosis was assessed by FACS analysis using Annexin V and PI staining. DMSO was used as control treatment. The plots are representative of three independent biological experiments.

Supplementary Table 1: Disease status, clinical parameters and cytogenetics of patients involved in this study

Sample. ID	M-Component (heavy chain)	M-Component (light chain)	Lines of treatment	FISH	Gender	Age
MM.01	IgA	kappa	0	1q gain, +5,+9,+11,+15	Male	65
MM.02	IgG	lambda	0	t(11;14), del(14q32)	Male	73
MM.03	IgG	lambda	0	t(14;16), 1q gain	Female	75
MM.04	IgG	kappa	0	+5, +11, +15, hyperdiploid	Female	69
MM.05	IgA	lambda	6	del(13q), 14q32 translocation	Male	74
MM.06	ND	kappa	3	14q32 translocation, +5,+9,+11,+15	Male	59
MM.07	IgG	lambda	2	del(13q), t(11;14)	Female	66
MM.08	IgA	lambda	3	del(13q), t(4;14), 1q gain	Male	59
MM.09	IgG	kappa	1	del(13q), del(17p), 1q gain, +11	Male	58
MM.10	IgG	kappa	2	del(17p), 14q32 translocation	Female	67
MM.11	IgG	kappa	5	del(13q), t(11;14), del(17p)	Female	67

MM: Multiple Myeloma; Ig: Immunoglobulin; del: Deletion; M: Male; F: Female.

Supplementary Table 2: Disease status, clinical parameters and purity of CD138+ cells from newly diagnosed MM patients used for combination treatments in this study

Patient No	M-Component (heavy chain)	M-Component (light chain)	% of CD138+ Purity
Patient 1	IgG	kappa	95%
Patient 2	IgG	kappa	99%
Patient 3	IgG	kappa	92%
Patient 4	IgG	kappa	98%
